# The role of cancer-associated fibroblasts, solid stress and other microenvironmental factors in tumor progression and therapy resistance

**DOI:** 10.1186/1475-2867-14-41

**Published:** 2014-05-16

**Authors:** Gvantsa Kharaishvili, Dana Simkova, Katerina Bouchalova, Mariam Gachechiladze, Nato Narsia, Jan Bouchal

**Affiliations:** 1 Laboratory of Molecular Pathology, Institute of Molecular and Translational Medicine, Faculty of Medicine and Dentistry, Palacky University, Olomouc, Czech Republic; 2 Department of Pediatrics and Laboratory of Experimental Medicine, Institute of Molecular and Translational Medicine, Faculty of Medicine and Dentistry, Palacky University, Olomouc, Czech Republic; 3 School of Cancer Sciences, University of Birmingham, Birmingham, UK

**Keywords:** Tumor microenvironment, Cancer associated fibroblasts, Solid stress, Interstitial fluid pressure, Therapy resistance

## Abstract

Tumors are not merely masses of neoplastic cells but complex tissues composed of cellular and noncellular elements. This review provides recent data on the main components of a dynamic system, such as carcinoma associated fibroblasts that change the extracellular matrix (ECM) topology, induce stemness and promote metastasis-initiating cells. Altered production and characteristics of collagen, hyaluronan and other ECM proteins induce increased matrix stiffness. Stiffness along with tumor growth-induced solid stress and increased interstitial fluid pressure contribute to tumor progression and therapy resistance. Second, the role of immune cells, cytokines and chemokines is outlined. We discuss other noncellular characteristics of the tumor microenvironment such as hypoxia and extracellular pH in relation to neoangiogenesis. Overall, full understanding of the events driving the interactions between tumor cells and their environment is of crucial importance in overcoming treatment resistance and improving patient outcome.

## Background

Tumor progression is partly a result of evolving crosstalk between different cell types within the tumor and its surrounding supportive tissue or tumor stroma [1]. Invasive tumor cells interact with the microenvironment and remodel it into a milieu supportive of tumor growth and tumor progression. The altered environment is recognizable under light microscope as desmoplasia and this is used for assessing invasion [2]. The importance of the microenvironment in tumor progression is shown using model systems. It has been shown on animal xenografts that injection of purified malignant epithelial cells results in the formation of histologically complex tumors, with 80% of the cells being stromal [3]. Further, injection of non-transformed mammary epithelial cells into irradiated mammary stromal fat pads, resulted in increased tumor growth compared to those injected into contralateral, non-irradiated mammary fat pads. Irradiated stromal cells altered the microenvironment and this resulted in tumor promotion [4]. Genetic alterations that initiate carcinoma, occur in the epithelium but events that promote tumor progression involve the stroma. In some cases, the trigger for neoplastic progression is speculated to come from signals within the stromal microenvironment [5]. Cancer cells release stroma-modulating growth factors such as fibroblast growth factor, members of the VEGF family, PDGF, EGFR ligands, interleukins, colony-stimulating factors, transforming growth factor β and many others [6]. These factors act in a paracrine manner, disrupt normal tissue homeostasis, resulting in stromal reactions such as angiogenesis and the inflammatory response [7,8].

### Fibroblasts in tumor progression and therapy resistance

Carcinoma associated fibroblasts (CAF) are believed to influence tumor behavior and outcome and thus knowledge of their biology is of importance to an overall understanding of cancer. CAFs are large, spindle-shaped mesenchymal cells that share characteristics with smooth muscle cells and fibroblasts [6]. They constitute a significant component of the stroma and represent the cells responsible for the change of extracellular matrix composition into one with increased amounts of collagens (desmoplastic response) [3]. Currently, no precise definition of CAFs exists because of the different cellular origin and markers expressed: CAFs are likely to derive from resident fibroblasts and marrow-derived mesenchymal precursor cells, whereas their generation through epithelial-mesenchymal transition (EMT) of tumor cells is more controversial [6,9,10]. CAFs are not only phenotypically but also functionally distinct from their normal counterparts and are identified immunocyto-/histochemically based on different markers such as α-smooth muscle actin (α-SMA), vimentin, desmin, fibroblast specific protein −1, PDGFR α and β, and fibroblast activation protein or their combinations; CAFs generally lose caveolin 1, PTEN, p21, or have mutated TP53 [11,12]. Some of these differences are reversible, whereas others persist when the fibroblasts are removed from the vicinity of the carcinoma cells. Their gene expression differences are due to epigenetic and genetic alterations [13,14] and relate to the stage of their differentiation [15]. The evidence above shows that CAFs, like tumor cells, are heterogenous, not only between different but also within the same type of cancer.

CAFs promote tumor progression in several ways such as secretion of multiple factors and MMPs, inducing stemness, EMT, epigenetic changes, etc. [11,16]. Recent breast cancer gene expression profiles of the stromal compartment, have revealed significantly different gene sets than normal mammary stroma, with increased cytokines, ECM molecules and proteases [17,18]. They alter the three dimensional ECM scaffold and support tumor cells that eventually metastasize and activate immune cells to enhance the ECM-degrading capacity [19]. Secreted ECM components such as tenascin reveal pro-migratory activity [20]. TGF-β induces HGF expression by fibroblasts and also induces the transition of fibroblasts to myofibroblasts by increasing α-SMA and tenascin C expression [21]. Gene expression changes, reported by Rajski et al., [22], which were induced by IGF-I in human breast fibroblasts, contained several soluble factors, such as periostin which is involved in bone metastasis and angiogenesis [23-25], tenascin, which enhances tumor cell proliferation [26], as well as LOXL1, a member of the lysyl oxidase family. LOXL1 like LOXL2, may act in the vicinity of epithelial cells during tissue remodeling and collagen cross-linking. LOXL2 has been reported to be involved in invasiveness [27] and specifically expressed by fibroblasts in tumor tissue [28]. The presence of these factors indicates that the IGF-I activated stroma enhances proliferation and the metastatic potential of the cancer cells. In this sense, periostin and tenascin C also activate developmental pathways for the viability of metastasis-initiating cells in the lungs [29]. In the pulmonary parenchyma, TGF-β3 stimulates myofibroblasts to produce periostin which binds stromal Wnt factors Wnt1 and Wnt3a for presentation to stem-like metastasis-initiating cells [30]. Myofibroblasts and the cancer cells themselves also produce tenascin C which promotes the intracellular functioning of the Wnt and Notch pathways [29]. The Wnt pathway is known to control stem cell maintenance in a variety of tissues [31] and tumors [32,30,34]. We previously identified another cancer associated extracellular matrix protein, asporin which may interact with the Wnt pathway [35-38]. Asporin was also identified as one of the most CAF-enriched molecules using gene ontology analysis and further suggested as a possible EMT marker due to colocalization with ZEB1 both in the stroma and epithelium of prostate cancer [39]. In breast and prostate carcinomas, mutations of critical tumor suppressor genes like PTEN and TP53 have been reported to occur in either epithelial or stromal cells in a mutually exclusive fashion [40,41]. Such findings indicate establishment of a vicious circle, in which mutations in the carcinoma drive alterations in the stroma that again promote carcinoma progression [40,41]. According to Dudley et al. [42], breast CAFs possess a nonmutated but functionally deficient form of p53 and TP53 mutation status may be a predictor of CAF-mediated chemoresistance [43]. The role of CAFs in chemo/endocrine and target resistance is well-reviewed in Mao et al. [11] using breast cancer as example. Besides the genetic mechanisms described above, there is also evidence supporting the involvement of epigenetic changes in the cancer stroma as a contributor to cancer progression. These include histone modifications and alterations in the expression of DNA methyltransferases, chromatin modifying factors and microRNAs [11]. Direct contact between stromal and tumor cells allows minor populations of the latter to evade chemotherapy. For example, adhesion of melanoma cells to fibroblast monolayers through N-cadherin activates AKT, which blocks BAD and significantly reduces the cytotoxic effects of the chemotherapy drug, cisplatin [44]. In summary, CAFs play a critical role in determining many aspects of tumor behavior and overall outcome.

### Matrix topology, stiffness and solid stress

Cancer initiation and progression are largely dependent on the physical and chemical features of the adjacent environment particularly matrix topology (architecture) and stiffness. These features are determined by the size of biopolymer (proteins, proteoglycans and glycosaminoglycans) fibers and the density of the fiber network [45]. Connective tissue is characterized by different fiber arrangements ranging from loose or random to highly aligned structures. ECM topology can provide important regulation of cell motility through physical cues that geometrically constrain adhesion sites to guide directional migration [46]. Cancer cells display aligning behavior, called contact guidance through which they actively remodel the ECM fibers surrounding a tumor, using contractile force to align the fibers perpendicularly to the tumor [47,45]. Dense fibrous collagen that is characteristic of breast cancer stroma forms radial patterns extending away from tumors [48]. On the other hand, the reticular orientation of the collagen matrix surrounding mammary glands may anchor and/or restrain cells. Thus, non-linear matrix reduces invasion while linear structure promotes it (reviewed in [46]).

Matrix concentration and post-translational modifications such as glycosylation and cross-linking (e.g. by LOXL1 and LOXL2) affect the mechanical properties, including visco-elasticity or stiffness (reviewed in [49]). Tumors are stiffer than their normal adjacent tissue. For example, healthy mammary gland is highly compliant (elastic modulus E = ~200 Pa), while the average tumor is over an order of magnitude stiffer (E = ~4,000 Pa). Both the tumor-surrounding stroma and vasculature exhibit increased stiffness (E = ~800–1,000 Pa and ~450 Pa, respectively) [47]. Increased matrix stiffness is also observed in fibrotic lungs, scar tissue and irradiated or aged tissue [50].

ECM topology and stiffness can influence mechanosensing and activate intracellular signaling to promote directional cell migration. Integrin receptors and the physical arrangement of adhesions could trigger orientation of the cytoskeleton, and matrix orientation can also stabilize leading edge protrusions to promote directionally persistent migration in which specific signaling pathways (via vinculin, talin, FAK, p130CAS and filamin A) are involved [45,46]. Cancer cells recognize an increase in ECM stiffness and respond by generating increased traction forces on their surroundings by regulating focal adhesion formation and growth factor signaling. For this purpose, the cell has several options: it can either force the network fibers apart and remodel the shape until it can pass through the pore, or tumor cell degrades the fiber matrix with the help of proteolytic enzymes (reviewed in [45]). This in turn, enhances growth, survival, and invasion of tumor cells by promoting focal adhesion maturation and signaling through actomyosin contractility [51]. Increased tumor tissue stiffness has been linked to tumor progression, direct stem cell differentiation, cell–cell and cell–matrix adhesion, hyaluronan synthesis, and expression of genes that play important roles in invasion and metastasis [52-54].

**Figure 1:**
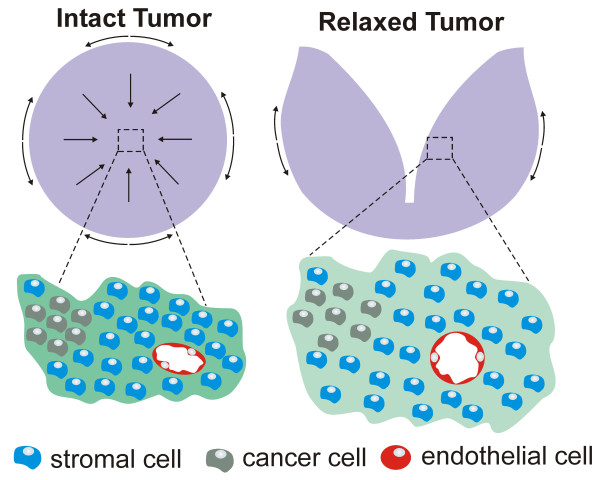
**Schematic of the biomechanical forces in the tumor microenvironment.** As tumor cells proliferate they sequentially create new solid material (i.e. cells and matrix components) which generate radial and circumferential solid stresses. In the tumor center, circumferential and radial stresses are compressive while in the periphery, radial stress is compressive and circumferential stress is tensile (direction indicated with arrows). Compressive stresses in the tumor interior squeeze tumor components, including lymphatic and blood vessels (note compressed lumen of blood vessel and high density of cells and extracellular matrix in dark green). After the tumor is cut and the stresses are released, the tumor interior decompresses (note extended lumen of blood vessel, relaxed cells and extracellular matrix in light green). Reproduced and modified with permission from Prof. Rakesh K. Jain [55].

Another important tumor characteristic is growth-induced solid stress (Figure 1). As tumor cells proliferate they sequentially create new solid material (i.e. cells and matrix components) which pushes against the surrounding tumor microenvironment. In normal tissue, the expansion of the tumor microenvironment is resisted by the enclosing microenvironment. However, cancer cells proliferate uncontrollably, ignoring contact inhibition and their expansion imposes elastic strain on the surrounding tumor microenvironment, storing stress through the deformation of compliant structures and collapsing more fragile structures, such as blood and lymphatic vessels (Figure 1). Interestingly, this solid stress is accumulated within the tumor and maintained even after the tumor is excised [55]. The known contribution of the ECM to solid stress includes both collagen and hyaluronan. Collagen resists tensile stress because it becomes stiffer as it is stretched. This finding is true for both capsular and interstitial collagen because the ECM in tumors is extensively cross-linked. Whereas hyaluronan resists compression, its negatively charged chains repel, owing to electrostatic repulsion and trap water, forming a poorly compressible matrix [55]. The compression of vessels by solid stress creates two potential barriers to drug delivery. First, the collapse of blood vessels hinders access of systemically administered drugs. This collapse might explain, in part, the fact that tumors with more ECM might be more resistant to treatment. For instance, pancreatic ductal adenocarcinomas, chondrosarcomas, and chordomas are rich in ECM and refractory to chemotherapy [56-58]. Second, the lack of lymphatic vessel function reduces drainage, leading to uniformly elevated interstitial fluid pressure (please see below). As a result, the transport of therapeutics, like antibodies and nanoparticles, is reduced because the dominant means of transport becomes diffusion which is a very slow process for large particles and macromolecules [59]. In this sense, decreasing solid stress by the inexpensive angiotensin inhibitor, losartan, enhances drug delivery and potentiates chemotherapy by decompressing tumor blood vessels [60].

### Immune cells in tumor microenvironment

Besides endothelial cells and fibroblasts, the tumor microenvironment also harbors innate and adaptive immune cells. It is a complex and highly dynamic system that should concomitantly work to eradicate a tumor. However, once a system is deformed, immunity becomes a benefit for the tumor and provides a very important cue to its development and progression [61]. The tumor-promoting effect of chronic inflammation has been reported many times [62,63]. However, how tumors promote inflammation and engage inflammatory cells in this process, is still being intensively studied. Macrophage density in the tumor generally negatively correlates with relapse-free and overall survival but localization also seems to be important. The association between TAM (tumor associated macrophage)-density and survival of cancer patients depends on the intratumoral or peritumoral macrophages counted [15]. TAMs promote the metastatic capacity of cancer cells in stromal or perivascular areas, while around the necrosis, in a hypoxic state, they stimulate angiogenesis [64]. A fibroblast secreted protein-1 (FSP1), also called S100A4 and mts1, is secreted by both fibroblasts and cancer cells, and also possibly by macrophages [65], making the environment more favourable to tumor progression by regulating inflammation and angiogenesis and promoting metastasis [66,67]. FSP1 is proangiogenic, possibly mediated either by the activation of plasminogen or through the transcriptional up-regulation of MMP13 [68]. Both of these proteinases play a role in endothelial cell invasion [5].

Immune cells, particularly macrophages and neutrophils are sources of chemokines, growth factors and proteases, as well as DNA-damaging reactive oxygen and nitrogen species. The gene expression profile of macrophages isolated from malignant tumors significantly differs from wound or resting peritoneal macrophage profile, with increased number of proliferation-associated genes [69]. Chemokines are produced not only by activated macrophages but by stromal and even cancer cells themselves. As an example, CXCL12 activates CXCR4 both on the surface of immune cells and on hematopoetic and endothelial precursors. The receptor is also expressed in some cancer cells. Accordingly, CXCL12 has several consequences: i) attraction of immune cells leading to tissue destruction, favoring invasion and metastasis; ii) promotion of growth and survival of cancer cells expressing the CXCR4 receptor; iii) recruitment of precursor cells for vasculogenesis; iv) activation of CXCR4 may also lead to greatly increased production of TNFα which itself exhibits other effects. Inflammatory cytokines, overexpressed by tumor cells recruit monocytes (macrophages), lymphocytes and neutrophils to tumor stroma, where they release VEGF, HGF, metalloproteinase 2 and interleukin 8 which affect endothelial cells and contribute to tumor progression [70]. Up-regulation of tumor-regulated interleukins 6 and1β is also associated with the inflammatory network, tumorigenesis, angiogenesis, and metastasis in breast, prostate, and pancreatic cancers [19]. Breast CAFs initiate and mediate tumorigenesis through a macrophage-recruitment inflammatory signature and is dependent on NF-kB signaling [71]. Recent studies demonstrate that colony stimulating factor (CSF)-1 which represents the main growth and differentiation factor for macrophages is overexpressed in breast, ovarian and prostate cancers [72]. Granulocyte CSF and granulocyte-macrophage CSF also contribute to progression of various cancers through recruitment of monocytes, macrophages and neutrophils into the tumor vicinity (reviewed in [6,73,74]).

### Tumor vasculature, hypoxia and interstitial fluid pressure

Tumor angiogenesis is an important factor in proliferation, metastasis, and drug sensitivity. Primary tumors without vasculature are small and dormant, while the growth of the tumor mass creates hypoxic conditions in the center of the tumor that induce expression of VEGF and subsequent tumor vascularization [75]. CAFs are also suggested to be an important source for growth factors and cytokines recruiting endothelial cells. These are involved in the establishment of the cancer stem cell niche and metastatic spread of tumor cells into distant organs [76]. Angiogenesis in malignant tumors, as measured by microvessel density correlates with clinicopathological factors or poor survival in many cancer types (reviewed in [77]). Tumor-endothelial cell interactions are mediated mostly by cell surface adhesion molecules (i.e. integrins, cadherins, immunoglobulins and selectins) [78,79]. CD34, CD31, and factor-VIII-related antigen are commonly used as tumor endothelial cell markers and microvessel density is determined by immunodetection of blood vessels with these markers. However, the markers identify not only neovessels but also pre-existing large ones [80]. Nestin has recently received attention as a marker of newly formed endothelial cells [81,82] and seems promising in the evaluation of neoangiogenesis of different tumors.

Hypoxia is also characteristic of abnormal tumor microenvironment that is intrinsically linked to the formation of neovasculature and is clinically associated with metastasis and poor patient outcome [83,84]. Diffusion-limited hypoxia is a consequence of tumor cells that are distant from the vascular supply. Such cells are exposed to prolonged or chronic hypoxia and tumor cells are viable in such environments for hours or a few days [85,86]. Hypoxia induces oncogene expression, enhances DNA mutation rate, and selects for cells with increased apoptotic thresholds [83,87]. Hypoxia drives tumor progression through increased matrix deposition, cross-linking and remodeling and, enhances collagen turnover and its fibril deposition [88]. Hypoxia-inducible factor (HIF)-1α plays an integral role in the body’s response to low oxygen. It is one of the primary genes involved in the homeostatic process which can increase vascularization in hypoxic areas. HIF-1α allows for survival and proliferation of cancer cells due to its angiogenic properties and its inhibition prevents the spread of cancer [89]. Many of the responses implicated in resistance to anti-angiogenic therapy may be mediated by HIF-1α activated genes [90]. The well-described inducers of EMT, Snail, Slug and Twist are themselves induced by hypoxia [91]. Hypoxia may also affect stem cells [92], and studies on this particular subpopulation of cells in tumors would be relevant to the metastatic process as cells surviving under hypoxic conditions become aggressive and pluripotent. Development of novel hypoxia-targeted therapies include bioreductive prodrugs, HIF-1 targeting, and genetic engineering of anaerobic bacteria [93].

Low extracellular pH is another consequence of the abnormal metabolism in the tumor and supportive factor for its progression. Products of anaerobic glycolysis - lactic acid and carbonic acid (produced by carbonic anhydrase from CO2 and H2O), are the known sources of H + ions in tumors [94,95]. The imbalance between increased production of H + ions and their reduced removal, lowers the extracellular pH in tumors. The mean pH profiles also decrease in tumors with increasing distance from nearest blood vessels. Low extracellular pH causes stress-induced alteration of gene expression, including the upregulation of VEGF and IL-8 in tumor cells *in vitro*[96]. Coordinated study of pH, pO2, and VEGF expression *in vivo*[97] indicated that in low pH or oxygenated regions, tissue pH, but not pO2, regulates VEGF promoter activity. Conversely, in hypoxic or neutral pH regions, tissue pO2 and not pH regulates VEGF expression [97]. Tissue pO2 and pH appear to regulate VEGF transcription in tumors independently. These data suggest that these key microenvironmental parameters in solid tumors regulate angiogenic factors in a complementary manner.

Another feature of the pathophysiology of the tumor microenvironment, is elevated interstitial fluid pressure (IFP) ranging from 10 to 100 mmHg [98,99] while IFP of normal tissue is around zero [100]. It is thought that the tumor vasculature is the driving force in increasing tumor IFP [101,102]. In contrast to normal vasculature which is characterized by dichotomous branching, tumor vasculature is unorganized and has trifurcations and branches with uneven diameters. Large inter-endothelial junctions, increased numbers of fenestrations, vesicles and vesico-vacuolar channels and a lack of normal basement membrane are often found in tumor vessels [103]. Due to ultrastructural alterations in the tumor vessel wall, vascular permeability in solid tumors is generally higher than that in various normal tissues [100]. Tumors also lack lymphatic vessels or the intratumoral vessels are non-functional [104,105] and as a result, excess fluid accumulates in the interstitium, extending the elastic ECM and elevating IFP. Using the model of IFP regulation Heldin et al. [106] showed that fibroblasts actively regulate the tension applied to the ECM through integrins which enable them to exert or modify tension on the collagen fibre network, thereby modulating the elasticity of the ECM in response to hyaluronan and proteoglycan expansion [106]. According to Oldberg et al. [107], inflammatory processes in carcinomas promote synthesis of collagen binding proteoglycan fibromodulin by stroma cells, leading to the formation of a dense and stiff collagen scaffold and a high IFP. Interstitial fluid pressure may have important clinical implications with regard to cancer therapy. Roh et al. [108] reported an inverse relationship between tumor IFP and tissue oxygenation and hypothesized that IFP may aid in predicting the efficacy of radiation therapy. Elevated tumor IFP can also act as a barrier to delivery of therapeutic agents, thereby reducing their efficacy. In this sense, multiple studies have demonstrated improved uptake of chemotherapeutic drugs following a reduction in tumor IFP [60,59,109,110].

## Conclusion

Interactions during which the tumor creates a microenvironment favourable for proliferation, for the recruitment of new blood vessels, and for the stimulation of the production of proteases that can degrade adjacent tissues, increase the likelihood of tumor development and invasion. Growing evidence points towards a key role of the multiple cellular and noncellular components of the tumor microenvironment such as cancer associated fibroblasts, immune and endothelial cells as well as matrix topology and stiffness, interstitial fluid pressure, growth induced solid stress, hypoxia and extracellular pH. Full understanding of all the events driving the interaction of tumor cells with their environment is of crucial importance in overcoming treatment resistance and in better patient outcome.

## Competing interests

The authors declare that they have no competing interests.

## Authors’ contributions

GK conceived the idea, did literature search and drafted the manuscript; DS did literature search and contributed in drafting the manuscript; MG, NN and KB were involved in discussion and drafting the manuscript; JB supervised the project, made substantial contributions to the concept and design of manuscript, and drafted the manuscript. All authors read and approved the final manuscript.

## References

[B1] Liotta LA Kohn EC The microenvironment of the tumour-host interface Nature 2001 411 375 379 10.1038/35077241 11357145

[B2] Koperek O Asari R Niederle B Kaserer K Desmoplastic stromal reaction in papillary thyroid microcarcinoma Histopathology 2011 58 919 924 10.1111/j.1365-2559.2011.03791.x 21477259

[B3] Dudley AC Shih SC Cliffe AR Hida K Klagsbrun M Attenuated p53 activation in tumour-associated stromal cells accompanies decreased sensitivity to etoposide and vincristine Br J Cancer 2008 99 118 125 10.1038/sj.bjc.6604465 18594537 PMC2453010

[B4] Barcellos-Hoff MH Ravani SA Irradiated mammary gland stroma promotes the expression of tumorigenic potential by unirradiated epithelial cells Cancer Res 2000 60 1254 1260 10728684

[B5] Dranoff G Cytokines in cancer pathogenesis and cancer therapy Nat Rev Cancer 2004 4 11 22 10.1038/nrc1252 14708024

[B6] Mueller MM Fusenig NE Friends or foes - bipolar effects of the tumour stroma in cancer Nat Rev Cancer 2004 4 839 849 10.1038/nrc1477 15516957

[B7] Baeriswyl V Christofori G The angiogenic switch in carcinogenesis Semin Cancer Biol 2009 19 329 337 10.1016/j.semcancer.2009.05.003 19482086

[B8] Chang HY Sneddon JB Alizadeh AA Sood R West RB Montgomery K Chi JT van de Rijn M Botstein D Brown PO Gene expression signature of fibroblast serum response predicts human cancer progression: similarities between tumors and wounds PLoS Biol 2004 2 E7 10.1371/journal.pbio.0020007 14737219 PMC314300

[B9] Kalluri R Zeisberg M Fibroblasts in cancer Nat Rev Cancer 2006 6 392 401 10.1038/nrc1877 16572188

[B10] Karnoub AE Dash AB Vo AP Sullivan A Brooks MW Bell GW Richardson AL Polyak K Tubo R Weinberg RA Mesenchymal stem cells within tumour stroma promote breast cancer metastasis Nature 2007 449 557 563 10.1038/nature06188 17914389

[B11] Mao Y Keller ET Garfield DH Shen K Wang J Stromal cells in tumor microenvironment and breast cancer Cancer Metastasis Rev 2013 32 303 315 10.1007/s10555-012-9415-3 23114846 PMC4432936

[B12] Ferrara N VEGF as a therapeutic target in cancer Oncology 2005 69 Suppl 3 11 16 16301831 10.1159/000088479

[B13] Herrera M Herrera A Dominguez G Silva J Garcia V Garcia JM Gomez I Soldevilla B Munoz C Provencio M Campos-Martin Y Garcia de Herreros A Casal I Bonilla F Pena C Cancer-associated fibroblast and M2 macrophage markers together predict outcome in colorectal cancer patients Cancer Sci 2013 104 437 444 10.1111/cas.12096 23298232 PMC7657228

[B14] Patocs A Zhang L Xu Y Weber F Caldes T Mutter GL Platzer P Eng C Breast-cancer stromal cells with TP53 mutations and nodal metastases N Engl J Med 2007 357 2543 2551 10.1056/NEJMoa071825 18094375

[B15] Baronzio G Freitas I Kwaan HC Tumor microenvironment and hemorheological abnormalities Semin Thromb Hemost 2003 29 489 497 14631549 10.1055/s-2003-44557

[B16] Elenbaas B Weinberg RA Heterotypic signaling between epithelial tumor cells and fibroblasts in carcinoma formation Exp Cell Res 2001 264 169 184 10.1006/excr.2000.5133 11237532

[B17] Singer CF Gschwantler-Kaulich D Fink-Retter A Haas C Hudelist G Czerwenka K Kubista E Differential gene expression profile in breast cancer-derived stromal fibroblasts Breast Cancer Res Treat 2008 110 273 281 10.1007/s10549-007-9725-2 17899370

[B18] Casey T Bond J Tighe S Hunter T Lintault L Patel O Eneman J Crocker A White J Tessitore J Stanley M Harlow S Weaver D Muss H Plaut K Molecular signatures suggest a major role for stromal cells in development of invasive breast cancer Breast Cancer Res Treat 2009 114 47 62 10.1007/s10549-008-9982-8 18373191

[B19] Schulz WA Burchardt M Cronauer MV Molecular biology of prostate cancer Mol Hum Reprod 2003 9 437 448 10.1093/molehr/gag064 12837920

[B20] Chauhan VP Stylianopoulos T Boucher Y Jain RK Delivery of molecular and nanoscale medicine to tumors: transport barriers and strategies Annu Rev Chem Biomol Eng 2011 2 281 298 10.1146/annurev-chembioeng-061010-114300 22432620

[B21] Untergasser G Gander R Lilg C Lepperdinger G Plas E Berger P Profiling molecular targets of TGF-beta1 in prostate fibroblast-to-myofibroblast transdifferentiation Mech Ageing Dev 2005 126 59 69 10.1016/j.mad.2004.09.023 15610763

[B22] Rajski M Zanetti-Dallenbach R Vogel B Herrmann R Rochlitz C Buess M IGF-I induced genes in stromal fibroblasts predict the clinical outcome of breast and lung cancer patients BMC Med 2010 8 1 10.1186/1741-7015-8-1 20051100 PMC2823652

[B23] Kharaishvili G Cizkova M Bouchalova K Mgebrishvili G Kolar Z Bouchal J Collagen triple helix repeat containing 1 protein, periostin and versican in primary and metastatic breast cancer: an immunohistochemical study J Clin Pathol 2011 64 977 982 10.1136/jclinpath-2011-200106 21742751

[B24] Sasaki H Yu CY Dai M Tam C Loda M Auclair D Chen LB Elias A Elevated serum periostin levels in patients with bone metastases from breast but not lung cancer Breast Cancer Res Treat 2003 77 245 252 10.1023/A:1021899904332 12602924

[B25] Schmidt-Hansen B Ornas D Grigorian M Klingelhofer J Tulchinsky E Lukanidin E Ambartsumian N Extracellular S100A4(mts1) stimulates invasive growth of mouse endothelial cells and modulates MMP-13 matrix metalloproteinase activity Oncogene 2004 23 5487 5495 10.1038/sj.onc.1207720 15122322

[B26] Ruiz C Huang W Hegi ME Lange K Hamou MF Fluri E Oakeley EJ Chiquet-Ehrismann R Orend G Growth promoting signaling by tenascin-C [corrected] Cancer Res 2004 64 7377 7385 10.1158/0008-5472.CAN-04-1234 15492259

[B27] Akiri G Sabo E Dafni H Vadasz Z Kartvelishvily Y Gan N Kessler O Cohen T Resnick M Neeman M Neufeld G Lysyl oxidase-related protein-1 promotes tumor fibrosis and tumor progression in vivo Cancer Res 2003 63 1657 1666 12670920

[B28] Hockel M Vaupel P Tumor hypoxia: definitions and current clinical, biologic, and molecular aspects J Natl Cancer Inst 2001 93 266 276 10.1093/jnci/93.4.266 11181773

[B29] Oskarsson T Massague J Extracellular matrix players in metastatic niches EMBO J 2012 31 254 256 10.1038/emboj.2011.469 22179697 PMC3261570

[B30] Malanchi I Santamaria-Martinez A Susanto E Peng H Lehr HA Delaloye JF Huelsken J Interactions between cancer stem cells and their niche govern metastatic colonization Nature 2012 481 85 89 22158103 10.1038/nature10694

[B31] Zeng YA Nusse R Wnt proteins are self-renewal factors for mammary stem cells and promote their long-term expansion in culture Cell Stem Cell 2010 6 568 577 10.1016/j.stem.2010.03.020 20569694 PMC2917779

[B32] Reya T Clevers H Wnt signalling in stem cells and cancer Nature 2005 434 843 850 10.1038/nature03319 15829953

[B33] Barker N Ridgway RA van Es JH van de Wetering M Begthel H van den Born M Danenberg E Clarke AR Sansom OJ Clevers H Crypt stem cells as the cells-of-origin of intestinal cancer Nature 2009 457 608 611 10.1038/nature07602 19092804

[B34] Kharaishvili G Simkova D Makharoblidze E Trtkova K Kolar Z Bouchal J Wnt signaling in prostate development and carcinogenesis Biomed Pap Med Fac Univ Palacky Olomouc Czech Repub 2011 155 11 18 10.5507/bp.2011.016 21475372

[B35] Turashvili G Bouchal J Baumforth K Wei W Dziechciarkova M Ehrmann J Klein J Fridman E Skarda J Srovnal J Hajduch M Murray P Kolar Z Novel markers for differentiation of lobular and ductal invasive breast carcinomas by laser microdissection and microarray analysis BMC Cancer 2007 7 55 10.1186/1471-2407-7-55 17389037 PMC1852112

[B36] Turtoi A Musmeci D Wang Y Dumont B Somja J Bevilacqua G De Pauw E Delvenne P Castronovo V Identification of novel accessible proteins bearing diagnostic and therapeutic potential in human pancreatic ductal adenocarcinoma J Proteome Res 2011 10 4302 4313 10.1021/pr200527z 21755970

[B37] Chouaib S Kieda C Benlalam H Noman MZ Mami-Chouaib F Ruegg C Endothelial cells as key determinants of the tumor microenvironment: interaction with tumor cells, extracellular matrix and immune killer cells Crit Rev Immunol 2010 30 529 545 10.1615/CritRevImmunol.v30.i6.30 21175416

[B38] Klee EW Bondar OP Goodmanson MK Dyer RB Erdogan S Bergstralh EJ Bergen HR 3rd Sebo TJ Klee GG Candidate serum biomarkers for prostate adenocarcinoma identified by mRNA differences in prostate tissue and verified with protein measurements in tissue and blood Clin Chem 2012 58 599 609 10.1373/clinchem.2011.171637 22247499 PMC3951013

[B39] Orr B Riddick AC Stewart GD Anderson RA Franco OE Hayward SW Thomson AA Identification of stromally expressed molecules in the prostate by tag-profiling of cancer-associated fibroblasts, normal fibroblasts and fetal prostate Oncogene 2012 31 1130 1142 10.1038/onc.2011.312 21804603 PMC3307063

[B40] Kurose K Gilley K Matsumoto S Watson PH Zhou XP Eng C Frequent somatic mutations in PTEN and TP53 are mutually exclusive in the stroma of breast carcinomas Nat Genet 2002 32 355 357 10.1038/ng1013 12379854

[B41] Shao R Bao S Bai X Blanchette C Anderson RM Dang T Gishizky ML Marks JR Wang XF Acquired expression of periostin by human breast cancers promotes tumor angiogenesis through up-regulation of vascular endothelial growth factor receptor 2 expression Mol Cell Biol 2004 24 3992 4003 10.1128/MCB.24.9.3992-4003.2004 15082792 PMC387763

[B42] D’Adamo DR Appraising the current role of chemotherapy for the treatment of sarcoma Semin Oncol 2011 38 Suppl 3 S19 S29 22055968 10.1053/j.seminoncol.2011.09.004

[B43] Sonnenberg M van der Kuip H Haubeis S Fritz P Schroth W Friedel G Simon W Murdter TE Aulitzky WE Highly variable response to cytotoxic chemotherapy in carcinoma-associated fibroblasts (CAFs) from lung and breast BMC Cancer 2008 8 364 10.1186/1471-2407-8-364 19077243 PMC2626600

[B44] Li G Satyamoorthy K Herlyn M N-cadherin-mediated intercellular interactions promote survival and migration of melanoma cells Cancer Res 2001 61 3819 3825 11325858

[B45] Brabek J Mierke CT Rosel D Vesely P Fabry B The role of the tissue microenvironment in the regulation of cancer cell motility and invasion Cell Commun Signal 2010 8 22 10.1186/1478-811X-8-22 20822526 PMC2941745

[B46] Petrie RJ Doyle AD Yamada KM Random versus directionally persistent cell migration Nat Rev Mol Cell Biol 2009 10 538 549 10.1038/nrm2729 19603038 PMC2752299

[B47] Kraning-Rush CM Reinhart-King CA Controlling matrix stiffness and topography for the study of tumor cell migration Cell Adh Migr 2012 6 274 279 10.4161/cam.21076 22863740 PMC3427241

[B48] Sidani M Wyckoff J Xue C Segall JE Condeelis J Probing the microenvironment of mammary tumors using multiphoton microscopy J Mammary Gland Biol Neoplasia 2006 11 151 163 10.1007/s10911-006-9021-5 17106644

[B49] Durand RE Raleigh JA Identification of nonproliferating but viable hypoxic tumor cells in vivo Cancer Res 1998 58 3547 3550 9721858

[B50] Mammoto A Mammoto T Kanapathipillai M Wing Yung C Jiang E Jiang A Lofgren K Gee EP Ingber DE Control of lung vascular permeability and endotoxin-induced pulmonary oedema by changes in extracellular matrix mechanics Nat Commun 2013 4 1759 23612300 10.1038/ncomms2774

[B51] Boghaert E Gleghorn JP Lee K Gjorevski N Radisky DC Nelson CM Host epithelial geometry regulates breast cancer cell invasiveness Proc Natl Acad Sci U S A 2012 109 19632 19637 10.1073/pnas.1118872109 23150585 PMC3511712

[B52] Levental KR Yu H Kass L Lakins JN Egeblad M Erler JT Fong SF Csiszar K Giaccia A Weninger W Yamauchi M Gasser DL Weaver VM Matrix crosslinking forces tumor progression by enhancing integrin signaling Cell 2009 139 891 906 10.1016/j.cell.2009.10.027 19931152 PMC2788004

[B53] Folgueras AR Pendas AM Sanchez LM Lopez-Otin C Matrix metalloproteinases in cancer: from new functions to improved inhibition strategies Int J Dev Biol 2004 48 411 424 10.1387/ijdb.041811af 15349816

[B54] Reinhart-King CA How matrix properties control the self-assembly and maintenance of tissues Ann Biomed Eng 2011 39 1849 1856 10.1007/s10439-011-0310-9 21491153 PMC3419599

[B55] Stylianopoulos T Martin JD Chauhan VP Jain SR Diop-Frimpong B Bardeesy N Smith BL Ferrone CR Hornicek FJ Boucher Y Munn LL Jain RK Causes, consequences, and remedies for growth-induced solid stress in murine and human tumors Proc Natl Acad Sci U S A 2012 109 15101 15108 10.1073/pnas.1213353109 22932871 PMC3458380

[B56] Provenzano PP Cuevas C Chang AE Goel VK Von Hoff DD Hingorani SR Enzymatic targeting of the stroma ablates physical barriers to treatment of pancreatic ductal adenocarcinoma Cancer Cell 2012 21 418 429 10.1016/j.ccr.2012.01.007 22439937 PMC3371414

[B57] Olive KP Jacobetz MA Davidson CJ Gopinathan A McIntyre D Honess D Madhu B Goldgraben MA Caldwell ME Allard D Frese KK Denicola G Feig C Combs C Winter SP Ireland-Zecchini H Reichelt S Howat WJ Chang A Dhara M Wang L Ruckert F Grutzmann R Pilarsky C Izeradjene K Hingorani SR Huang P Davies SE Plunkett W Egorin M Inhibition of Hedgehog signaling enhances delivery of chemotherapy in a mouse model of pancreatic cancer Science 2009 324 1457 1461 10.1126/science.1171362 19460966 PMC2998180

[B58] Chauhan VP Martin JD Liu H Lacorre DA Jain SR Kozin SV Stylianopoulos T Mousa AS Han X Adstamongkonkul P Popovic Z Huang P Bawendi MG Boucher Y Jain RK Angiotensin inhibition enhances drug delivery and potentiates chemotherapy by decompressing tumour blood vessels Nat Commun 2013 4 2516 24084631 10.1038/ncomms3516 PMC3806395

[B59] Honn KV Tang DG Adhesion molecules and tumor cell interaction with endothelium and subendothelial matrix Cancer Metastasis Rev 1992 11 353 375 10.1007/BF01307187 1423822

[B60] Horino Y Takahashi S Miura T Takahashi Y Prolonged hypoxia accelerates the posttranscriptional process of collagen synthesis in cultured fibroblasts Life Sci 2002 71 3031 3045 10.1016/S0024-3205(02)02142-2 12408871

[B61] Hu M Yao J Cai L Bachman KE van den Brule F Velculescu V Polyak K Distinct epigenetic changes in the stromal cells of breast cancers Nat Genet 2005 37 899 905 10.1038/ng1596 16007089

[B62] Castellsague X Bosch FX Munoz N Environmental co-factors in HPV carcinogenesis Virus Res 2002 89 191 199 10.1016/S0168-1702(02)00188-0 12445659

[B63] Nelson WG DeWeese TL DeMarzo AM The diet, prostate inflammation, and the development of prostate cancer Cancer Metastasis Rev 2002 21 3 16 10.1023/A:1020110718701 12400993

[B64] Lewis CE Pollard JW Distinct role of macrophages in different tumor microenvironments Cancer Res 2006 66 605 612 10.1158/0008-5472.CAN-05-4005 16423985

[B65] Inoue T Plieth D Venkov CD Xu C Neilson EG Antibodies against macrophages that overlap in specificity with fibroblasts Kidney Int 2005 67 2488 2493 10.1111/j.1523-1755.2005.00358.x 15882296

[B66] Goetz JG Minguet S Navarro-Lerida I Lazcano JJ Samaniego R Calvo E Tello M Osteso-Ibanez T Pellinen T Echarri A Cerezo A Klein-Szanto AJ Garcia R Keely PJ Sanchez-Mateos P Cukierman E Del Pozo MA Biomechanical remodeling of the microenvironment by stromal caveolin-1 favors tumor invasion and metastasis Cell 2011 146 148 163 10.1016/j.cell.2011.05.040 21729786 PMC3244213

[B67] Fukumura D Jain RK Tumor microenvironment abnormalities: causes, consequences, and strategies to normalize J Cell Biochem 2007 101 937 949 10.1002/jcb.21187 17171643

[B68] Semenza GL Targeting HIF-1 for cancer therapy Nat Rev Cancer 2003 3 721 732 10.1038/nrc1187 13130303

[B69] De Wever O Nguyen QD Van Hoorde L Bracke M Bruyneel E Gespach C Mareel M Tenascin-C and SF/HGF produced by myofibroblasts in vitro provide convergent pro-invasive signals to human colon cancer cells through RhoA and Rac FASEB J 2004 18 1016 1018 15059978 10.1096/fj.03-1110fje

[B70] Zumsteg A Christofori G Corrupt policemen: inflammatory cells promote tumor angiogenesis Curr Opin Oncol 2009 21 60 70 10.1097/CCO.0b013e32831bed7e 19125020

[B71] Kobayashi Y The role of chemokines in neutrophil biology Front Biosci 2008 13 2400 2407 10.2741/2853 17981721

[B72] Lin CW Shen SC Ko CH Lin HY Chen YC Reciprocal activation of macrophages and breast carcinoma cells by nitric oxide and colony-stimulating factor-1 Carcinogenesis 2010 31 2039 2048 10.1093/carcin/bgq172 20876703

[B73] Coussens LM Werb Z Inflammation and cancer Nature 2002 420 860 867 10.1038/nature01322 12490959 PMC2803035

[B74] Balkwill F Cancer and the chemokine network Nat Rev Cancer 2004 4 540 550 10.1038/nrc1388 15229479

[B75] Egeblad M Littlepage LE Werb Z The fibroblastic coconspirator in cancer progression Cold Spring Harb Symp Quant Biol 2005 70 383 388 10.1101/sqb.2005.70.007 16869775 PMC2580828

[B76] Li L Neaves WB Normal stem cells and cancer stem cells: the niche matters Cancer Res 2006 66 4553 4557 10.1158/0008-5472.CAN-05-3986 16651403

[B77] Meert AP Paesmans M Martin B Delmotte P Berghmans T Verdebout JM Lafitte JJ Mascaux C Sculier JP The role of microvessel density on the survival of patients with lung cancer: a systematic review of the literature with meta-analysis Br J Cancer 2002 87 694 701 10.1038/sj.bjc.6600551 12232748 PMC2364252

[B78] Buess M Rajski M Vogel-Durrer BM Herrmann R Rochlitz C Tumor-endothelial interaction links the CD44(+)/CD24(−) phenotype with poor prognosis in early-stage breast cancer Neoplasia 2009 11 987 1002 19794958 10.1593/neo.09670 PMC2745665

[B79] Heldin CH Rubin K Pietras K Ostman A High interstitial fluid pressure - an obstacle in cancer therapy Nat Rev Cancer 2004 4 806 813 10.1038/nrc1456 15510161

[B80] Ishiwata T Matsuda Y Naito Z Nestin in gastrointestinal and other cancers: effects on cells and tumor angiogenesis World J Gastroenterol 2011 17 409 418 10.3748/wjg.v17.i4.409 21274370 PMC3027007

[B81] Teranishi N Naito Z Ishiwata T Tanaka N Furukawa K Seya T Shinji S Tajiri T Identification of neovasculature using nestin in colorectal cancer Int J Oncol 2007 30 593 603 17273760

[B82] Kolar Z Ehrmann J Jr Turashvili G Bouchal J Mokry J A novel myoepithelial/progenitor cell marker in the breast? Virchows Arch 2007 450 607 609 10.1007/s00428-007-0403-x 17429688

[B83] Lunt SJ Chaudary N Hill RP The tumor microenvironment and metastatic disease Clin Exp Metastasis 2009 26 19 34 10.1007/s10585-008-9182-2 18543068

[B84] Grum-Schwensen B Klingelhofer J Berg CH El-Naaman C Grigorian M Lukanidin E Ambartsumian N Suppression of tumor development and metastasis formation in mice lacking the S100A4(mts1) gene Cancer Res 2005 65 3772 3780 10.1158/0008-5472.CAN-04-4510 15867373

[B85] Sutherland RM Franko AJ On the nature of the radiobiologically hypoxic fraction in tumors Int J Radiat Oncol Biol Phys 1980 6 117 120 10.1016/0360-3016(80)90215-1 6988374

[B86] Devarajan-Ketha H Craig TA Madden BJ Robert Bergen H 3rd Kumar R The sclerostin-bone protein interactome Biochem Biophys Res Commun 2012 417 830 835 10.1016/j.bbrc.2011.12.048 22206666 PMC3259242

[B87] Duff MD Mestre J Maddali S Yan ZP Stapleton P Daly JM Analysis of gene expression in the tumor-associated macrophage J Surg Res 2007 142 119 128 10.1016/j.jss.2006.12.542 17597158

[B88] Helmlinger G Sckell A Dellian M Forbes NS Jain RK Acid production in glycolysis-impaired tumors provides new insights into tumor metabolism Clin Cancer Res 2002 8 1284 1291 11948144

[B89] Schauer IG Sood AK Mok S Liu J Cancer-associated fibroblasts and their putative role in potentiating the initiation and development of epithelial ovarian cancer Neoplasia 2011 13 393 405 21532880 10.1593/neo.101720 PMC3084616

[B90] Bagley RG Endosialin: from vascular target to biomarker for human sarcomas Biomark Med 2009 3 589 604 10.2217/bmm.09.54 20477527

[B91] Sun S Ning X Zhang Y Lu Y Nie Y Han S Liu L Du R Xia L He L Fan D Hypoxia-inducible factor-1alpha induces Twist expression in tubular epithelial cells subjected to hypoxia, leading to epithelial-to-mesenchymal transition Kidney Int 2009 75 1278 1287 10.1038/ki.2009.62 19279556

[B92] Keith B Simon MC Hypoxia-inducible factors, stem cells, and cancer Cell 2007 129 465 472 10.1016/j.cell.2007.04.019 17482542 PMC3150586

[B93] Sun JD Liu Q Wang J Ahluwalia D Ferraro D Wang Y Duan JX Ammons WS Curd JG Matteucci MD Hart CP Selective tumor hypoxia targeting by hypoxia-activated prodrug TH-302 inhibits tumor growth in preclinical models of cancer Clin Cancer Res 2012 18 758 770 10.1158/1078-0432.CCR-11-1980 22184053

[B94] Garin-Chesa P Old LJ Rettig WJ Cell surface glycoprotein of reactive stromal fibroblasts as a potential antibody target in human epithelial cancers Proc Natl Acad Sci U S A 1990 87 7235 7239 10.1073/pnas.87.18.7235 2402505 PMC54718

[B95] Pouyssegur J Dayan F Mazure NM Hypoxia signalling in cancer and approaches to enforce tumour regression Nature 2006 441 437 443 10.1038/nature04871 16724055

[B96] Xu L Fukumura D Jain RK Acidic extracellular pH induces vascular endothelial growth factor (VEGF) in human glioblastoma cells via ERK1/2 MAPK signaling pathway: mechanism of low pH-induced VEGF J Biol Chem 2002 277 11368 11374 10.1074/jbc.M108347200 11741977

[B97] Erler JT Weaver VM Three-dimensional context regulation of metastasis Clin Exp Metastasis 2009 26 35 49 10.1007/s10585-008-9209-8 18814043 PMC2648515

[B98] Nathanson SD Nelson L Interstitial fluid pressure in breast cancer, benign breast conditions, and breast parenchyma Ann Surg Oncol 1994 1 333 338 10.1007/BF03187139 7850532

[B99] Milosevic M Fyles A Hedley D Pintilie M Levin W Manchul L Hill R Interstitial fluid pressure predicts survival in patients with cervix cancer independent of clinical prognostic factors and tumor oxygen measurements Cancer Res 2001 61 6400 6405 11522633

[B100] Erler JT Cawthorne CJ Williams KJ Koritzinsky M Wouters BG Wilson C Miller C Demonacos C Stratford IJ Dive C Hypoxia-mediated down-regulation of Bid and Bax in tumors occurs via hypoxia-inducible factor 1-dependent and -independent mechanisms and contributes to drug resistance Mol Cell Biol 2004 24 2875 2889 10.1128/MCB.24.7.2875-2889.2004 15024076 PMC371100

[B101] Lunt SJ Kalliomaki TM Brown A Yang VX Milosevic M Hill RP Interstitial fluid pressure, vascularity and metastasis in ectopic, orthotopic and spontaneous tumours BMC Cancer 2008 8 2 10.1186/1471-2407-8-2 18179711 PMC2245966

[B102] Jain RK Tong RT Munn LL Effect of vascular normalization by antiangiogenic therapy on interstitial hypertension, peritumor edema, and lymphatic metastasis: insights from a mathematical model Cancer Res 2007 67 2729 2735 10.1158/0008-5472.CAN-06-4102 17363594 PMC3022341

[B103] Winkler F Kozin SV Tong RT Chae SS Booth MF Garkavtsev I Xu L Hicklin DJ Fukumura D di Tomaso E Munn LL Jain RK Kinetics of vascular normalization by VEGFR2 blockade governs brain tumor response to radiation: role of oxygenation, angiopoietin-1, and matrix metalloproteinases Cancer Cell 2004 6 553 563 15607960 10.1016/j.ccr.2004.10.011

[B104] Padera TP Kadambi A di Tomaso E Carreira CM Brown EB Boucher Y Choi NC Mathisen D Wain J Mark EJ Munn LL Jain RK Lymphatic metastasis in the absence of functional intratumor lymphatics Science 2002 296 1883 1886 10.1126/science.1071420 11976409

[B105] Leu AJ Berk DA Lymboussaki A Alitalo K Jain RK Absence of functional lymphatics within a murine sarcoma: a molecular and functional evaluation Cancer Res 2000 60 4324 4327 10969769

[B106] Fukumura D Xu L Chen Y Gohongi T Seed B Jain RK Hypoxia and acidosis independently up-regulate vascular endothelial growth factor transcription in brain tumors in vivo Cancer Res 2001 61 6020 6024 11507045

[B107] Oldberg A Kalamajski S Salnikov AV Stuhr L Morgelin M Reed RK Heldin NE Rubin K Collagen-binding proteoglycan fibromodulin can determine stroma matrix structure and fluid balance in experimental carcinoma Proc Natl Acad Sci U S A 2007 104 13966 13971 10.1073/pnas.0702014104 17715296 PMC1955775

[B108] Roh HD Boucher Y Kalnicki S Buchsbaum R Bloomer WD Jain RK Interstitial hypertension in carcinoma of uterine cervix in patients: possible correlation with tumor oxygenation and radiation response Cancer Res 1991 51 6695 6698 1742744

[B109] Vlahovic G Ponce AM Rabbani Z Salahuddin FK Zgonjanin L Spasojevic I Vujaskovic Z Dewhirst MW Treatment with imatinib improves drug delivery and efficacy in NSCLC xenografts Br J Cancer 2007 97 735 740 10.1038/sj.bjc.6603941 17712313 PMC2360385

[B110] Pietras K Rubin K Sjoblom T Buchdunger E Sjoquist M Heldin CH Ostman A Inhibition of PDGF receptor signaling in tumor stroma enhances antitumor effect of chemotherapy Cancer Res 2002 62 5476 5484 12359756

